# What Is the Sweetest UPR Flavor for the β-cell? That Is the Question

**DOI:** 10.3389/fendo.2020.614123

**Published:** 2021-01-19

**Authors:** Alina Lenghel, Alina Maria Gheorghita, Andrei Mircea Vacaru, Ana-Maria Vacaru

**Affiliations:** Institute for Cellular Biology and Pathology “Nicolae Simionescu”, Bucharest, Romania

**Keywords:** unfolded protein response (UPR) pathway, β-cell proliferation, β-cell dedifferentiation, immune attack, heterogeneity

## Abstract

Unfolded protein response (UPR) is a process conserved from yeasts to mammals and, based on the generally accepted dogma, helps the secretory performance of a cell, by improving its capacity to cope with a burden in the endoplasmic reticulum (ER). The ER of β-cells, “professional secretory cells”, has to manage tremendous amounts of insulin, which elicits a strong pressure on the ER intrinsic folding capacity. Thus, the constant demand for insulin production results in misfolded proinsulin, triggering a physiological upregulation of UPR to restore homeostasis. Most diabetic disorders are characterized by the loss of functional β-cells, and the pathological side of UPR plays an instrumental role. The transition from a homeostatic to a pathological UPR that ultimately leads to insulin-producing β-cell decay entails complex cellular processes and molecular mechanisms which remain poorly described so far. Here, we summarize important processes that are coupled with or driven by UPR in β-cells, such as proliferation, inflammation and dedifferentiation. We conclude that the UPR comes in different “flavors” and each of them is correlated with a specific outcome for the cell, for survival, differentiation, proliferation as well as cell death. All these greatly depend on the way UPR is triggered, however what exactly is the switch that favors the activation of one UPR as opposed to others is largely unknown. Substantial work needs to be done to progress the knowledge in this important emerging field as this will help in the development of novel and more efficient therapies for diabetes.

## Introduction

Recently, increased stress of the endoplasmic reticulum (ER), or ER stress, has emerged as a critical regulator of transcription and translation events in diabetes (1–5). The ER supports correct protein folding that is essential to maintain protein homeostasis and cell survival; however, this process is remarkably sensitive as even minute modifications in the cellular milieu can result in protein misfolding (6, 7). Following nutrient stimulation, freshly transcribed insulin mRNA translated in the ER drives a 10-fold increase in insulin synthesis that represents about 50% of the total protein production by the β-cells (7, 8). This massive synthesis and its variations result in a constant hassle of the ER. To deal with this challenge, β-cells continuously supervise protein folding in the ER through a well conserved mechanism, the unfolded protein response (UPR) (7, 9).

Over the next sections, we will present how UPR is actively present during important stages of β-cell existence in physiological and pathological circumstances. We will focus on processes that are relevant for the development of potential therapies that target UPR. As such, we will address the role of UPR in proliferation, inflammation/inflammatory attack and dedifferentiation of β-cells.

## UPR And Development Of Diabetes

UPR is a cellular process consisting of an intricate network of transducers and downstream target genes ensuring correct protein folding in the ER. UPR comprises of three major sensors: Protein kinase RNA-like endoplasmic reticulum kinase (PERK), endoribonuclease/kinase inositol-requiring enzyme 1 (IRE1, or ERN1), and activating transcription factor 6 (ATF6) (10, 11). These factors are localized in the ER membrane and they are able to sense and monitor through their luminal domains the status of protein folding in the ER (12–17). If an accumulation of unfolded proteins occurs, these transducers signal *via* their cytosolic domains either by direct targeted catalytic activities or by specific post-translational modulation. The precise mechanism that triggers UPR is still under debate (18–22), and most probably there is not a single mechanism involved, but rather the multiple concerted action of several ones, depending on cell type (23–25). The downstream effectors converge at the nucleus and induce UPR targets, finally restoring homeostasis *via* various processes described below.

PERK, upon oligomerization followed by autophosphorylation (26–28), phosphorylates the translation initiation factor 2 (eIF2α) inducing inhibition of mRNA translation through activation of a signaling cascade, thus reducing the ER protein load, and increasing ATF4 translation (13, 27, 29, 30). This results in overexpression of chaperones, antioxidant genes, but also of proapoptotic genes, such as CHOP, GADD34, ATF3 and TRB3 that contribute to β-cell apoptosis during terminal UPR (31, 32). eIF2α has a central role in stress management, being also targeted by other kinases in response to various kinds of stresses (30, 33–36). This signaling cascade converging on eIF2α phosphorylation followed by ATF4 activation is an adaptive pathway for cellular homeostasis restoration commonly known as Integrated Stress Response (ISR) (37, 38).

IRE1 possesses both kinase and endoribonuclease activities. When UPR is induced, dimerization and trans-autophosphorylation of IRE1 activates its RNase domain and results in splicing of *Xbp1* pre-mRNA and overexpression of XBP1s, a transcription factor that induces genes-encoding chaperones, ER-associated protein degradation (ERAD), and lipid biosynthetic enzymes (12, 17, 39–41). Additionally, IRE1 presents a nonspecific RNase activity responsible for degradation of mRNAs from ER vicinity, thus reducing import of proteins into the ER (26, 42, 43). During increased stress, the kinase activity of IRE1 is activated and initiates the apoptosis cascade mediated by signal-regulating kinase 1 (ASK1)/cJun amino terminal kinase (JNK) (44).

Upon UPR activation, ATF6 is translocated to the Golgi apparatus, where it is processed by Site-1 and Site-2 proteases (S1P/S2P) (45, 46). Once the cytosolic fragment (nuclear ATF6, or nATF6) is generated, it travels to the nucleus and induces transcription of UPR target genes (47–49). Alone or in combination with XBP1s, nATF6 acts on increasing synthesis of chaperones to aid with the misfolded proteins, of proteins involved in lipid synthesis to increase ER volume, and of genes responsible for the ERAD pathway. They work for restoring homeostasis by modulating the amount of ER-mediated production of ER lipids and proteins necessary to accommodate variable requirements of ER protein folding and other functions in response to physiological and pathological conditions. If any of these mechanisms fail, the ER homeostasis is lost, a stressed UPR is induced and that ultimately results in cell apoptosis (11, 32, 50–52).

In diabetes, overactivation of UPR leads to phosphorylation of IRE1, which results in degradation of proinsulin mRNA (53–55) activation of JNK pathway, and splicing of XBP1 mRNA. XBP1s by itself or in cooperation with ATF6 induces expression of various ER chaperons, such as Herp1, EDEM, HRD1, p58IPK, and ERAD proteins, followed by swelling of the ER. Moreover, CHOP mRNA expression is induced by both ATF4 and XBP1s (5). By depleting CHOP in various diabetes models results in improved β-cell function and survival (56), although in NOD mice it is not the case (57). Surprisingly, TUDCA was able to increase the expression of Atf6 and XBP1 and increased β-cells survival, reduced islet inflammation and thus lower diabetes incidence in mouse models of diabetes (58). Therefore, in diabetes, the erroneous expression of ER chaperones may be responsible for the predisposition of the β-cell to a terminal UPR that culminates with cell death induced by CHOP.

PERK-eiF2α-ATF4 and IRE1-XBP1s/ATF6 arms of the UPR are activated differently by glucose. Surprisingly, low glucose concentrations result in maximal activation of the first arm, while protein synthesis, ATP levels and the concentration of Ca^2+^ in the ER are low, whereas the second arm is inactive. The response to high glucose concentration is the rapid inhibition of the ISR, the splicing of Xbp1 pre-mRNA and subsequent upregulation of XBP1s together with the downstream target genes to accommodate increased ER machinery load. Finely adjusting this adaptive response is indispensable to preserve the identity and function of β-cell (59).

## UPR Is Very Dynamic And Drives Heterogenic Insulin Expression

Xin and collaborators have shown that in healthy human subjects, β-cells go through different active states to accommodate insulin requirements that are characterized by different levels of UPR and insulin gene expression. They show that the transition between an active and prolonged insulin secretory state results in induction of a stressed UPR that diminishes the levels of secreted insulin. After a certain time, the UPR of the β-cell recovers at a basal level and the cells restart the production of insulin. They describe several cyclical individual states of β-cell stress in correlation with insulin secretion, and low apoptosis and dedifferentiation markers (60). UPR was induced in a subpopulation of β-cells that express low insulin levels (INS^low^UPR^hi^) suggesting they represent a state of recovery from stress. Another population of β-cells was characterized by INS^hi^UPR^low^ and most likely represents a state of active production and secretion (60). Importantly, the insulin protein amounts are not always correlated with the mRNA levels. Apparently, in pancreases of type 1 diabetes (T1D) patients insulin protein levels were very low; nevertheless proinsulin and INS mRNA were still detected (61). It is not clear if this occurs due to dedifferentiation of β-cells or because more precursors of β-cells arise (61, 62). The characterization of different populations of β-cells based on their UPR and insulin levels is crucial in diabetes. It is important to know how these populations change during the disease progression, and where and when to intervene therapeutically to recover insulin homeostasis.

## β-cells With Active UPR Proliferate

One of the questions that puzzled scientists referred to how is β-cell mass regulation maintained? While it is already established that stem cells drive regeneration of tissues with fast turnover, such as skin, gut and blood, a stem cell pool has not yet been characterized for the pancreatic islets (63). Multiple studies demonstrated that β-cell mass adjusts in a dynamic way, in correlation with increased metabolic demand, or upon injury. Under most conditions, the major driver of postnatal islet cell expansion is the proliferation of already present β-cells (64).

Several studies suggested that UPR activation *in vivo* drives β-cell proliferation. Hodish and collaborators showed that overexpression of mutant proinsulin is correlated with both UPR activation and islet size increase (65). Inhibiting expression of PERK in adult mice resulted in increased proliferation of β-cells (66). In addition, another study established that ATF6 pathway that acts in response to the loss of PERK is regulating the pro-proliferative UPR mechanism rather than PERK. By using different murine models of diabetes (glucose-induced hyperglycemia mouse model; db/db mice and Akita mice) as well as β-cells isolated from pancreatic donors, they argue that mild UPR drives ATF6-induced proliferation of β-cells based on the insulin requirement. Moreover, they show that inhibition of ATF6 and IRE1 pathways reduce glucose-induced β-cell proliferation *in vitro.* However, chemical chaperones abrogated the proliferative effect on the β-cells (63). Human β-cells are less likely to respond well to stimulation, as they have a lower basal proliferation than mouse cells (67, 68). Importantly, there are studies showing proliferation of β-cells from donors upon UPR stimulation (63). A thorough study that characterized various β-cells subpopulation from healthy subjects showed that the majority of proliferating cells displayed low insulin expression correlated with activated UPR, with more proliferating cells in G1S cell cycle phase rather than in G2M (60).

## UPR And Inflammation In β-cells

The questions raised here are: does the dysregulated UPR from β-cells facilitate the immune attack, or vice-versa, the cytokines secreted by the immune cells induced upregulation of the UPR in β-cells, rendering them more susceptible to apoptosis? Many studies proved that both are true and mostly interdependent.

### Inflammatory Environment Triggers UPR Activation

Recent work shows the importance of inflammation for UPR induction and β-cell fate in various diabetes contexts, especially in T1D (9, 69). There, the progressive invasion of inflammatory cells, like T-cells, macrophages, dendritic cells, and natural killer cells within the islets leads to insulitis (9, 70–72). Due to insulitis, the access of numerous proinflammatory molecules and reactive oxygen species (ROS) to β-cells, like interleukin-1β (IL1β), TNF, IFN-γ, IL17, and NO, is facilitated as these molecules are secreted by the invading immune cells. This results in apoptosis of β-cells (73, 74). Death of β-cells driven by cytokines entails induction of various transcription factors (NF-κB and STAT1), JNK, which in conjunction with a stressed UPR, end with activation of mitochondrial pathway of apoptosis (73, 75, 76). Moreover, upon stimulation by pro-inflammatory cytokines, β-cells start expressing and secreting more cytokines and chemokines, resulting in a cross-talk between the immune cells and the β-cells (77, 78). As a consequence, many T cells get infiltrated into the islets and cause destruction of the β-cells initiating diabetes (73, 79, 80).

The low grade inflammation present in the pancreas of T2D patients is responsible for recruitment of macrophages in the vicinity of the islets creating a pro-inflammatory milieu and inducing UPR (81, 82). A recent study provided direct evidence for the role of ER stress-induced inflammation in T2D. It revealed that by blocking IL23 and IL24, proinflammatory cytokines upregulated in the islets of T2D patients, the authors were able to partially decrease oxidative stress, UPR induction and restore glucose tolerance in obese mice. In addition, after reducing ROS with IL22, the improved UPR stress and β-cell function re-established glucose homeostasis (83).

### UPR Facilitates Inflammatory Attack of β-Cells

This research topic got attention, as emerging data connects inflammatory responses to UPR in β-cells *via* the regulator of inflammation, NFκB (84, 85). Additionally, XBP1 seems to exert both pro- and anti-inflammatory effects in β-cells depending on the context established by the anti-apoptotic/anti-oxidative reaction as opposed to the pro-inflammatory response (47). Moreover, CHOP was shown to have a pro-inflammatory role in various disease models, upregulating pro-inflammatory cytokines (such as IL1β, IL8) and chemokines (CCL2) in several tissues (86, 87). However, it is not clear how CHOP activates NF-κB. In β-cells, studies show that the transcription factor NF-κB is able to modulate the UPR upon activation by pro-inflammatory cytokines (88, 89). Reciprocally, the UPR was found to induce NF-κB activity in correlation with inflammatory responses, resulting in increased apoptosis and overexpression of cytokines and chemokines that may be responsible for β-cell death (74, 89, 90).

## UPR In Dedifferentiation Of β-cells

It has been established that every cell from any organism, β-cells included, are derived by differentiation from embryonic stem cells (91). Differentiation toward β-cells involves synchronized and rigorously controlled induction/downregulation of certain transcription factors and effectors in a timely manner  (59). Importantly, cellular differentiation is not unidirectional (92). Recent data has shown that specific factors can induce mature β-cells to lose their identity and phenotype and backslide to an under-differentiated state, or in a progenitor-like condition. This phenomena is called dedifferentiation and has been involved in the pathology of diabetes (93–96), being a significant contributor of the reduction of functional β-cell mass (62, 97). Dedifferentiation of β-cells is characterized by reduced expression of β-cell-specific genes, that include essential transcription factors, insulin, genes responsible for glucose metabolism, genes required for protein processing and genes of the secretory pathway, accompanied by induction of genes that are usually repressed or lowly expressed in mature β-cell, such as the embryonic endocrine progenitor genes. Expression of these later genes is found in diabetic animals, in the islets (59, 62, 94). The mechanisms involved in the dedifferentiation process are still under investigation and here, we will underline some possible implications of UPR.

In a recent study, Zhu and collaborators have demonstrated that overexpression of miR24 reduced ER stress-induced β-cells apoptosis and blocked INS mRNA degradation, though it induced dedifferentiation of β-cells (98). MiR24 was found to inactivate the IRE1 sensor. Importantly, they speculated that one of the downstream effectors of IRE1 was CHOP. As ATF4 was not affected by miR24, the assumption was that CHOP was not upregulated *via* the PERK/ATF4 pathway. Surprisingly, they demonstrated that XBP1s, effector downstream of IRE1, is responsible for the apoptosis of β-cells under terminal ER stress (98). In a T1D model, work from Engin’s group has shown that downregulating IRE1 before insulitis appearance results in temporary dedifferentiation of β-cells proved to be beneficial as it made β-cells more resistant to the immune attack (99). These dedifferentiated β-cells expressed lower levels of autoantigens and of MHC class I molecules and upregulated their immune inhibitory markers (99, 100). It became apparent that interference with TGFβ signaling resulted in induction of several markers of β-cells maturation (101, 102) and reversed dedifferentiation (103). The E3 ligase Hsd1 and the cofactor Sel1L represent well-conserved ERAD machinery (104) that has recently been linked to the preservation of β-cells identity *via* inhibition TGFβ pathway, while their survival and proliferation were not affected (105).

## Conclusions and Perspectives

The correlation between activation of UPR and their insulin gene expression was shown to divide β-cells into several populations that evolve from dynamic insulin secretion states to a stress recovery state when insulin production is decreased (60). This is an important aspect when developing new therapies that have a scope to mimic the heterogeneity of the β-cells. Strategies for regenerating β-cells should consider the importance to reintroduce these differences in the newly-emerged cells.

In the β-cells, there are functions of UPR that are ER stress-independent. Hassler and collaborators, by using mice with inducible β-cells-specific deletion of IRE1α, established the importance IRE1α/XBP1s pathway for glucose-stimulated insulin synthesis. The study revealed that this pathway regulates recruitment and structure of the ribosome, translation of pro-insulin mRNA, cleavage of the signal peptide, and inhibition of oxidative/inflammatory stress. Early activation of this UPR pathway appears to happen separately of ER stress and precedes the glucose-stimulated insulin synthesis (47).

Prolonged upregulation of a stressed UPR induces apoptosis, a process that involves JNK activation by the pro-inflammatory cytokines that act upon pancreatic β-cells through the progression of diabetes. It is not clear though the definitive mechanisms that pro-inflammatory cytokines use to induce IRE1α and JNK in human β-cells. This will help build new strategies to inhibit the UPR-driven pro-apoptotic signals without disturbing the other homeostatic functions (70).

We previously defined the ER stress as a stressed/terminal/decompensated UPR, the ultimate stage of UPR, with no recovery, when the cell enters the apoptosis pathway. One of the questions here is how do the cells get to the terminal UPR. What are the stages that precede it and can be targeted through therapies? We and others have challenged two different models for UPR activation: 1) the “rheostat model”, when the UPR sensors and targets get activated in a concerted fashion and they are correlated with the level of stress - the more stress, the higher the UPR. This model is described by different subclasses that are characterized by the type and extent of the stress (52, 106). 2) Some stressors induce a UPR subclass that causes a specific outcome, physiological or pathological. This could vary from “adaptive” to the “terminal” UPR, when the cell sees many facets of UPR through a combination of different arms that are activated differently. These are such broad definitions, and future thorough characterizations are necessary to address the precise role of each of them in the β-cells fate.

Possible therapies that use UPR for restoring β-cells homeostasis should consider the existing β-cell stress level and action inside a narrow safe range to overcome the excess and cell death. One possibility is employing agents that recover β-cell stress from terminal stage to the adaptive stage thus to facilitate the increase of β-cell mass through the mild stress. Therefore, there is a need for more tools for measuring and modulating β-cell stress *in vivo*.

## Author Contributions

AL, AG, AMV, and AnaMV read and reviewed the final version of the work, and participated in bibliographical research and design. AL assisted in the writing. AnaMV designed the concept and wrote the manuscript. All authors contributed to the article and approved the submitted version.

## Funding

The research leading to these results has received funding from the NO Grants 2014-2021, under Project contract no. 21/2020 (RO-NO-2019-0544; BETAUPREG to AnaMV) and from a project co-financed by European Regional Development Fund through the Competitiveness Operational Program 2014–2020 (POC-A.1-A.1.1.4-E-2015, ID: P_37_668; DIABETER) and by Romanian Academy.

## Conflict of Interest

The authors declare that the research was conducted in the absence of any commercial or financial relationships that could be construed as a potential conflict of interest.
